# Community psychological and behavioural responses to coronavirus disease 2019 over one year of the pandemic in 2020 in Hong Kong

**DOI:** 10.1038/s41598-021-00616-9

**Published:** 2021-11-18

**Authors:** Qiuyan Liao, Jingyi Xiao, Justin Cheung, Tiffany W. Y. Ng, Wendy W. T. Lam, Michael Y. Ni, Benjamin J. Cowling

**Affiliations:** 1 School of Public Health, LKS Faculty of Medicine, The University of Hong Kong, Pok Fu Lam, Hong Kong Special Administrative Region China; 2 World Health Organization Collaborating Centre for Infectious Disease Epidemiology and Control, School of Public Health, The University of Hong Kong, Pok Fu Lam, Hong Kong Special Administrative Region China; 3 Jockey Club Institute of Cancer Care, LKS Faculty of Medicine, The University of Hong Kong, Pok Fu Lam, China; 4 State Key Laboratory of Brain and Cognitive Sciences, The University of Hong Kong, Pok Fu Lam, Hong Kong Special Administrative Region China; 5 Healthy High Density Cities Lab, HKUrbanLab, The University of Hong Kong, Pok Fu Lam, Hong Kong Special Administrative Region China; 6 Laboratory of Data Discovery for Health, Hong Kong Science and Technology Park, Sha Tin, Hong Kong Special Administrative Region China

**Keywords:** Infectious diseases, Psychology

## Abstract

Monitoring community psychological and behavioural responses to coronavirus disease 2019 (COVID-19) is important for informing policy making and risk communication to sustain public compliance with challenging precautionary behaviours and mitigating the psychological impacts. Monthly telephone-based cross-sectional surveys in January–April 2020 and then weekly surveys from May through December 2020 were conducted to monitor changes in public risk perception of COVID-19, personal efficacy in self-protection, confidence in government’s ability to control the pandemic, precautionary behaviours, perceived impact of precautionary behaviours, psychological fatigue and distress in Hong Kong, and examine their inter-relationships. While worry about contracting COVID-19 increased, personal efficacy and confidence in government declined as the community incidence of COVID-19 increased. The public maintained high compliance with most precautionary behaviours throughout but relaxed behaviours that were more challenging when disease incidence declined. Public confidence in government was persistently low throughout, of which, a lower level was associated with more psychological fatigue, lower compliance with precautionary behaviours and greater psychological distress. Perceived greater negative impact of precautionary behaviours was also associated with more psychological fatigue which in turn was associated with relaxation of precautionary behaviours. Female, younger and unemployed individuals reported greater psychological distress throughout different stages of the pandemic. Risk communication should focus on promoting confidence in self-protection and pandemic control to avoid helplessness to act when the pandemic resurges. Policy making should prioritize building public trust, enhancing support for sustaining precautionary behaviours, and helping vulnerable groups to adapt to the stress during the pandemic.

## Introduction

The coronavirus disease 2019 (COVID-19) pandemic has caused enormous global disruption. Hong Kong with population 7.5 million had reported 12,147 cases of COVID-19 including 213 fatal cases as of 13 September 2021^1^. Although the number of COVID-19 cases to date has been relatively small, Hong Kong had experienced a number of surges in transmission in 2020^1^. Prior to the availability of an effective vaccine, public engagement with public health and social measures (PHSMs) was critical for pandemic control^2^. Public perception of their vulnerability to and the severity of the disease, personal confidence in self-protection and trust in the government in pandemic control were documented to be important determinants of compliance with PHSMs during COVID-19 pandemic^3–6^. However, these existing studies^3–6^ covered only a short period of the initial pandemic stage. There remained limited understanding about to what extent people could sustain their engagement with PHSMs as the threat of the pandemic continued, and what contributed to the public’s sustained engagement with PHSMs.

On the other hand, the pandemic and its control measures have caused considerable psychological impact in different populations^7–11^. For instance, studies reported a prevalence of 35% of psychological distress in Chinese adults^12^, a prevalence of around 15%, 8% and 5% of moderate, moderately severe and severe depression symptoms, respectively, in US adults^13^, and a prevalence of 27% of mental distress of clinical significance in UK adults^14^ during an early stage of the pandemic. The psychological impact caused by the pandemic can be more severe among populations who have fewer resources or lower personal capacity to cope with the changing situation. There is consistent evidence that female gender, younger age, lower income and being unemployed are associated with greater psychological distress during the pandemic^8,10,11,15^. Most relevant studies were conducted during the initial stage of the pandemic or when the countries were lockdown^7–10,12–15^. Studies conducted over a relatively longer period indicated that some groups of the population may recover from the initial psychological impact when local transmission of disease slowed down or social distancing measures were relaxed while others’ psychological symptoms may last for a longer period^16–18^. How the repeated resurgence of the pandemic would affect the mental health of population of different sociodemographic strata remained underexplored.

### Theoretical framework

According to the Transactional Theory of Stress and Coping (TTSC)^19^, the primary stressor (e.g. COVID-19) stimulates stress appraisal which comprises evaluation of potential threat, harm caused by the stressor and individual resources or capacity to manage the stress. The stress appraisal subsequently motivates various behavioural coping strategies. TTSC also proposes that appraisal of the primary stressor (i.e., the pandemic) and the secondary stressors (e.g., the control measures) could cause psychological impact^19^. Stress results from the interaction between personal resource (internal and external) and the situation^20^. People who perceive lower capacity or fewer resources to cope with the changing situation would suffer from greater psychological distress. In addition, according to the Model of Self-regulation (MSR)^21^, coping with stress can also be viewed as a self-regulatory process through which individuals take actions to cope with the changing situation to achieve their personal goals (e.g., self-protection or pandemic control). This subsequently generates a feedback route through which individuals evaluate whether their personal goals are achieved with the effort input. If negative feedbacks (e.g., the pandemic repeatedly surges and negative impacts of the coping strategies) are received, it will cause feeling of self-regulation failure and fatigue (e.g., pandemic fatigue)^22,23^ which will in turn demotivates existing coping effort (e.g., compliance with precautionary behaviours).

### Study objectives and hypotheses

This was a repeated cross-sectional study to monitor the changes of public stress appraisal including COVID-19 risk perception, personal efficacy and confidence in government’s pandemic control, behavioural coping (i.e., precautionary behaviours) and psychological distress over one year of the pandemic in 2020 in Hong Kong. We also aimed to examine the interrelationships among appraisal of stress and precautionary behaviours, adoption of precautionary behaviours and psychological distress, and to identify sociodemographic factors associated with increased vulnerability to psychological distress throughout different stages of the pandemic. Based on existing literature and our theoretical framework, we hypothesized that: H1. Public risk perception of COVID-19 (i.e., perceived personal susceptibility, worry, perceived severity), personal precautionary behaviours against COVID-19 and psychological distress would increase while perceived personal efficacy in self-protection and confidence in government’s pandemic control would decline as local incidence of COVID-19 increased, and vice versus; H2. Females, younger adults, adults with lower educational attainment and unemployed adults would have greater psychological distress than their counterparts; H3. Greater risk perception of COVID-19, personal efficacy and confidence in government would be associated with more adoption of precautionary behaviours, while greater risk perception of COVID-19, and lower personal efficacy and confidence in government would be associated with greater psychological distress. To test the self-regulatory process, in the later stage of the pandemic when people had adopted physical distancing measures for some time, but the pandemic remained unresolved, we also hypothesized that: H4. Negative reappraisal of personal effort (for achieving personal goals) including reduced personal efficacy, reduced confidence in government and perceived more negative impacts of the physical distancing measures would be associated with a feeling of self-regulatory failure and thereby more feeling of psychological fatigue and grater psychological distress; in addition, psychological fatigue due to negative appraisal of their self-regulatory effort would in turn be associated with lower compliance with precautionary behaviours.

## Materials and methods

We followed the STROBE checklist (https://www.strobe-statement.org) to report this study.

### Study design and sampling

Repeated cross-sectional surveys were conducted from January 2020, immediately before any cases of COVID-19 were reported in Hong Kong, through to December 2020. Surveys were conducted monthly from January to April 2020 with a sample size of approximately 1000 in each round, and survey frequency increased to weekly thereafter with the sample size alternating between 500 and 1000 in each round. The sample size in each round allows estimate of the prevalence of specific population properties with margin of errors of 3–5%. Adults (aged ≥ 18 years) were recruited using random digit dialling of landline and mobile phones with a ratio of 1:1 randomly generated by computer. Participants were included if they could speak Cantonese and were capable of answering a telephone interview. One adult within each selected household whose birthday was soonest for residential phone calls or the owners of the mobile phone numbers for mobile phone calls were invited to complete the telephone interview. Calls were made during non-working hours as well as working hours to avoid oversampling of non-working groups. Participants were newly recruited in each round. Each round was conducted for a period of 3–5 days and each interview was kept within 15 min. Informed consent was obtained from all participants. This study was conducted in accordance with the Declaration of Helsinki and Good Clinical Practices. The study received ethical approval from the Institutional Review Board of the University of Hong Kong (Reference No.: UW 20-095).

### Study instruments

The questionnaire for each survey round included the same core study measures throughout, with additional measures included in a subset of survey weeks to ensure feasible length of each questionnaire while enabling examination of a wide spectrum of influences on stress appraisal, precautionary behaviours and mental health outcomes. Core study measures included perceived personal susceptibility from COVID-19, worry about contacting COVID-19, perceived severity of COVID-19, personal efficacy in self-protection, confidence in the government’s ability to control the pandemic, personal hygiene practices (PHP), physical distancing behaviours (PDB) and psychological distress. Measures of COVID-19-related risk perceptions, beliefs and precautionary behaviours were similar to that used in our surveys during the outbreak of severe respiratory syndrome (SARS) in 2003^24^, influenza A(H1N1) pandemic in 2009–2010^25,26^ and outbreaks of avian influenza A(H7N9) in 2013–2015^27^. Psychological distress was assessed with the 4-item Patient Health Questionnaire (PHQ-4) that comprises two items from the Generalized Anxiety Disorder-7 (GAD-7) and two items from the PHQ-9 depression measure^28,29^. Each item of the PHQ-4 was rated on a Likert scale ranging from 0 (‘not at all’) to 3 (‘nearly every day’) with a higher score indicating greater distress. A summed scores of  ≥ 3 of the two items from GAD-7 was used for assessing probable anxiety^30^ while that of the two items from PHQ-9 was used for assessing probable depression^31^.The total score of all four items of PHQ-4 was also calculated to indicate overall psychological distress^28,29^. In Round 25 (Sep. 2020) and Round 33 (Nov. 2020), perceived negative impacts of physical distancing measures and ‘psychological fatigue’ with the pandemic were measured. According to the World Health Organization (WHO), psychological fatigue with the pandemic is defined as feeling of distress or frustration due to “sustained and unresolved adversity”^23^. Based on this definition, psychological fatigue was operationalized as feeling of tiredness with COVID-19 and emotional exhaustion (helplessness and frustration) regarding pandemic control in this study. Detailed descriptions of the study measures were provided in Suppmentary Table 1.

### Data analysis

To test H1, means or proportions of core study measures were calculated and weighted to the Hong Kong census data on age and sex in 2019 and plotted by survey round and against incidence of COVID-19 in Hong Kong.

For H2, to ensure sufficient sample sizes for the comparisons of mental health outcomes by sociodemographic strata, survey rounds were combined to represent five phases of the COVID-19 epidemic in Hong Kong in 2020: Phase 1 from January to April 2020 (Round 1–4) was an early phase of the epidemic characterized by mainly cases imported from mainland China and overseas; Phase 2 from May to June (Round 5–12) was a quiescent phase characterized by merely sporadic cases; Phase 3 from July to August (Round 13–20) included the first major community epidemic of COVID-19 characterized by widespread local-acquired infections; Phase 4 from August to November (Round 21–33) included a period when community incidence was lower again; and Phase 5 from November to December 2020 (Round 34–39) included the second major community epidemic of COVID-19. Weighted proportions of probable anxiety and probable depression by major sociodemographic strata were compared and plotted by the five phases. Logit regression enabled examination of associations of probable anxiety and probable depression with major sociodemographic factors adjusting for epidemic phase.

Testing H3 and H4 involved testing two structural equation models (SEM) based on TTSC^19^ and MSR^21^ to understand the relationships among stress appraisal, behavioural coping and the self-regulation process during the pandemic. Model 1 was to test H3 in which stress appraisal involving COVID-19 risk perceptions, personal efficacy and confidence in government were hypothesized to influence PHP, PDB and psychological distress. Socio-demographic factors including age, sex, educational attainment and occupational status that indicated resources and capacity to cope with stress were hypothesized to be associated with stress appraisal in this model. Model 2 was aimed to test a feedback process of the self-regulatory effort (H4), in which lower personal efficacy, lower confidence in government and perceived more negative impacts of the physical distancing measures were hypothesized to be associated with greater feeling of fatigue and psychological distress while more psychological fatigue was in turn associated with lower adoption of precautionary behaviours. Multiple-item measures were treated as latent variables for which confirmatory factor analysis (CFA) was first run to test their correlations before they were included into the full SEM. Robust maximum likelihood estimator was used to estimate the SEM models. Multiple model fit indices including CFI, TLI, SRMR and RMSEA were used to assess model fit with CFI > 0.90, TLI > 0.90, SRMR < 0.08 and RMSEA < 0.08 indicating a good fit of the model to the data. The CFA and SEM were running using Mplus 7.3 for Windows which enables handling missing data using full information maximum likelihood.

## Results

### Participants

**Figure 1:**
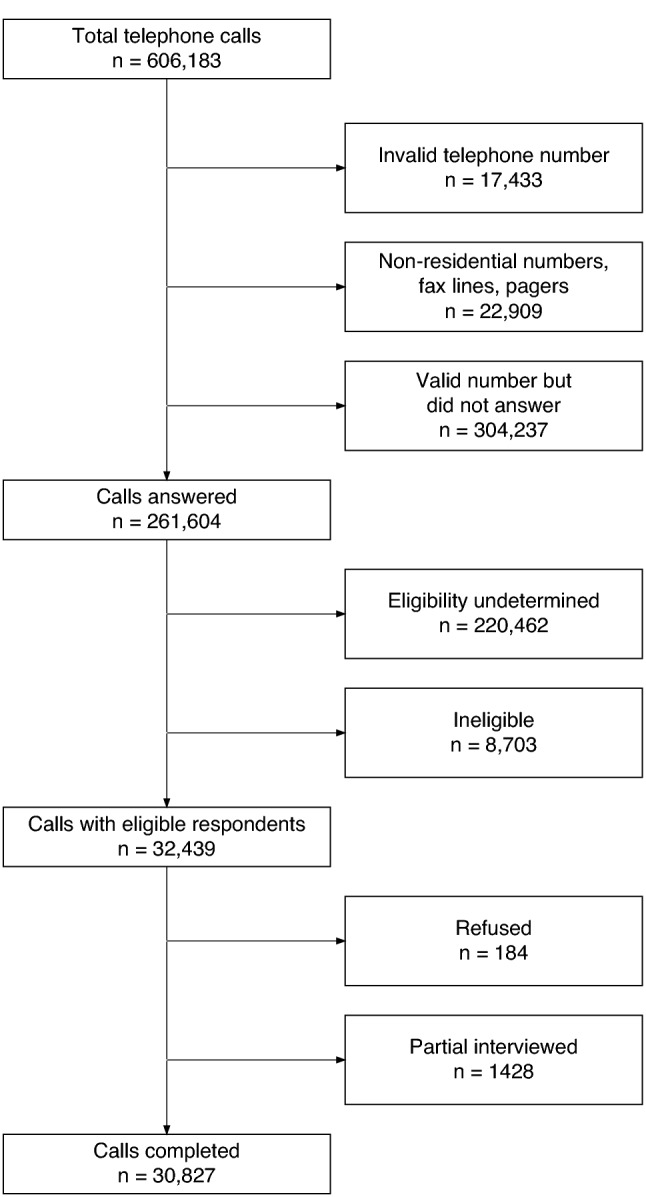
The flow chat of subject recruitment and interview.

A total of 39 rounds of telephone surveys were conducted from January to December 2020. Overall, a total of 32,439 subjects answered the calls and were screened to be eligible, of whom, 30,827 (95.0%) completed the interview (Fig. 1).

### Temporal changes in stress appraisal, precautionary behaviours and psychological distress

**Figure 2:**
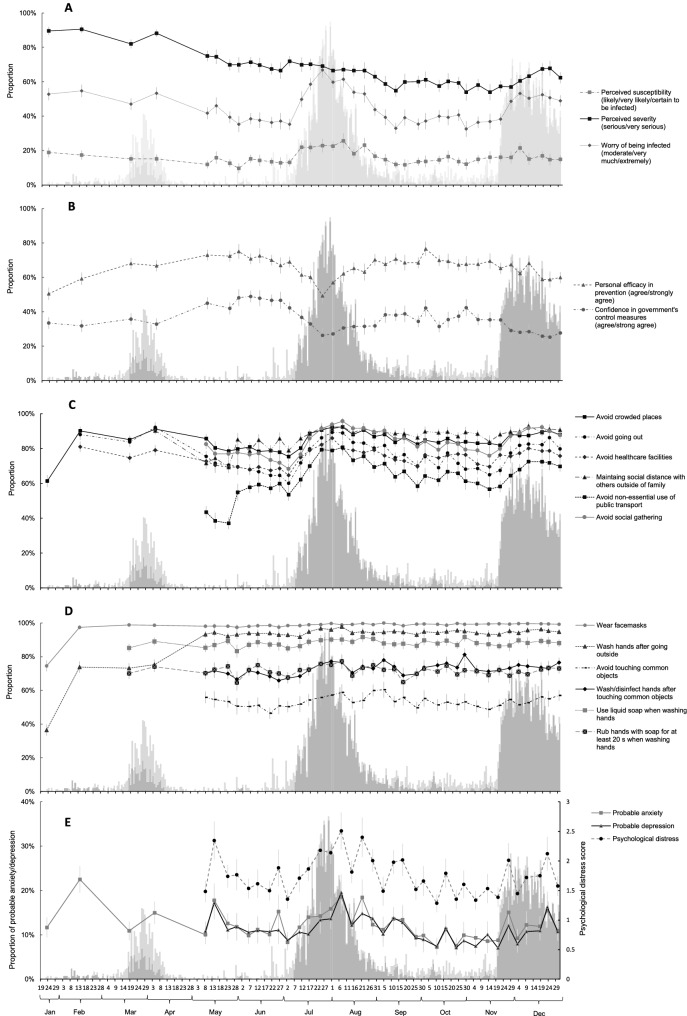
Daily confirmed cases of COVID-19 in Hong Kong and the temporal changes in risk perception of COVID-19 (**A**), personal efficacy in the prevention of COVID-19 and confidence in government’s control measures (**B**), probable anxiety, probable depression and overall psychological distress (**C**), adoption of physical distancing behaviours (**D**) and adoption of personal hygiene practices (**E**).

Figure 2 shows the temporal changes in stress appraisal, precautionary behaviours and psychological distress. Prevalence of some stress appraisal variables and precautionary behaviours measures in Round 1–3 had been previously reported^2^ but were also included here for comparison. Perceiving vulnerability to COVID-19 were relatively higher in the first two rounds (17–18%) but declined (10–15%) thereafter with only slight increases when the community incidence of COVID-19 was higher (Panel A). Prevalence of reporting at least moderate worry about contacting COVID-19 was around 30–40% when the disease incidence was low but increased to more than 60% when the disease incidence reached a peak (Panel A). Around 90% perceived the severity of COVID-19 to be severe or very severe initially, which declined to 50–60% in late 2020 except for a slight increase in December 2020 when there was a small bounce in number of death cases due to COVID-19 (Panel A). Around 60–70% believed in personal ability in self-protection against COVID-19 when the disease incidence was low, which declined to 50% when locally acquired cases increased rapidly in the community (Panel B). However, Confidence in government was low, being ~ 30–50% throughout and declined to below 30% when disease incidence increased (Panel B).

Prevalence of avoiding crowded places, avoiding social gathering and maintaining appropriate social distance with others outside of family were maintained at a level of more than 70–80% throughout and reached to more than 90% when disease incidence was high (Panel C). Avoiding going out, avoiding healthcare facilities and avoiding non-essential use of public transport (e.g., for leisure activity) were seen to decline more when disease incidence was lower but increased to 70–80% or more when disease incidence was higher (Panel C).

The prevalence of wearing facemasks when going outside had reached to almost 100% since Round 2 when COVID-19 cases were first reported in Hong Kong (Panel D). Prevalence of washing hands after going outside increased dramatically within the first four months of the pandemic and had maintained at a level of more than 90% ever since (Panel D). Around 80% and 70% reported using liquid soap and rubbing hands with soap for at least 20 s, respectively, when washing hands throughout (Panel D). Around 50–60% reported avoiding touching common objects in public places but 70–80% would wash or disinfect hands immediately after touching public objects (Panel D).

Probable anxiety reached a peak of ~ 20% when COVID-19 cases were first reported in Hong Kong which declined thereafter to around 10% with additional peaks detected in Round 6, Round 18 and Round 38, respectively, when community incidence increased or when social distancing measures were prolonged even if community incidence substantially declined (Panel E). The temporal trends of probable depression and overall psychological distress score were similar (Panel E).

### Psychological distress by socio-demographic strata

**Figure 3:**
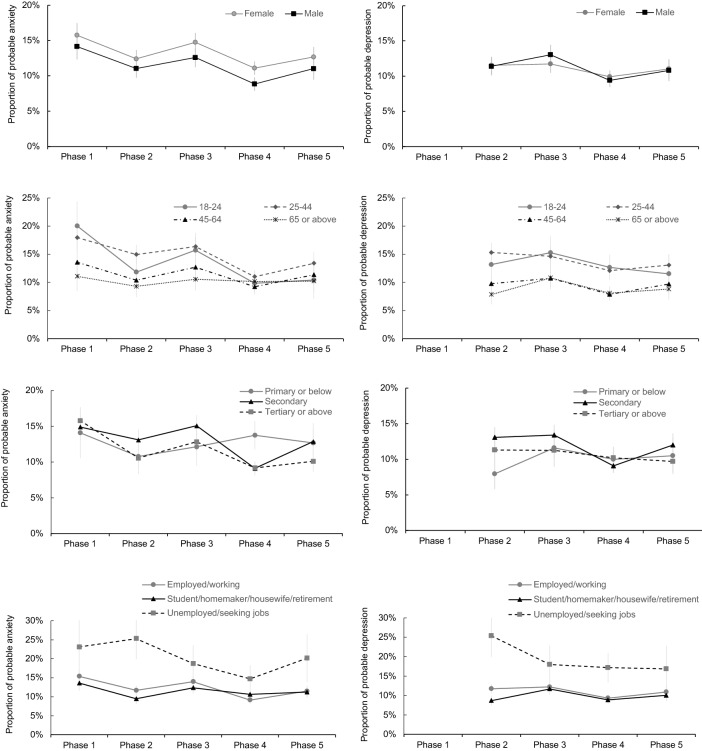
Proportions of respondents who had probable anxiety and probable depression by sex, age, educational attainment and occupation.

Females were more likely to have probable anxiety but not probable depression while younger, less educated and unemployed participants were more likely to have both probable anxiety and probable depression than their counterparts (Table 1). Throughout the five phases of the pandemic, the prevalence of probable anxiety was consistently higher in females than males, in participants aged 18–24 years and 25–44 years than those who were older and in participants who were unemployed or seeking jobs (Fig. 3). Participants aged 18–24 years and 25–44 years and those who were unemployed or seeking jobs were also found to have a higher prevalence of probable depression throughout the five phases of the pandemic (Fig. 3).

**Table 1:** Associations of probable anxiety and probable depression with socio-demographics and phases of pandemic.

	Odds ratio (95% confidence interval)	
	Probable anxiety	Probable depression
Sex (male vs. female)	0.86 (0.79–0.94)^b^	1.02 (0.93–1.13)
**Age (years)**		
18–24	1.00	1.00
25–34	1.14 (0.94–1.39)	1.16 (0.95–1.41)
35–44	1.04 (0.85–1.27)	0.86 (0.69–1.06)
45–54	0.78 (0.63–0.96)^a^	0.60 (0.48–0.74)^c^
55–64	0.67 (0.54–0.83)^c^	0.55 (0.44–0.69)^c^
≥ 65	0.58 (0.45–0.74)^c^	0.51 (0.39–0.66)^c^
**Educational attainment**		
Primary or below	1.00	1.00
Secondary	0.77 (0.67–0.88)^c^	0.89 (0.76–1.04)
Tertiary or above	0.60 (0.51–0.71)^c^	0.69 (0.57–0.82)^c^
**Occupation**		
Executive and professional	1.00	1.00
Clerical and service worker	1.00 (0.89–1.13)	1.07 (0.93–1.23)
Production worker	1.06 (0.90–1.25)	1.09 (0.91–1.31)
Student	1.10 (0.87–1.39)	1.10 (0.86–1.42)
Homemaker/housewife	1.10 (0.94–1.29)	1.15 (0.96–1.38)
Retirement	1.04 (0.87–1.25)	1.26 (0.92–1.38)
Unemployed or seeking jobs	1.84 (1.54–2.20)^c^	2.09 (1.72–2.54)^c^
**Epidemic phase**		
Phase 1 (initial phase with mainly imported cases)	1.00	–
Phase 2 (relative quiescent phase)	0.74 (0.65–0.85)^c^	1.00
Phase 3 (first major community epidemic)	0.88 (0.77–1.00)	1.10 (0.97–1.24)
Phase 4 (phase with relatively low community incidence)	0.62 (0.54–0.70)^c^	0.82 (0.73–0.92)^b^
Phase 5 (second major community epidemic)	0.75 (0.65–0.86)^c^	0.95 (0.83–1.09)

^a^*p* < 0.05; ^b^*p* < 0.01; ^c^*p* < 0.001.

### Stress appraisal, precautionary behaviours, psychological distress and the self-regulation process

**Figure 4:**
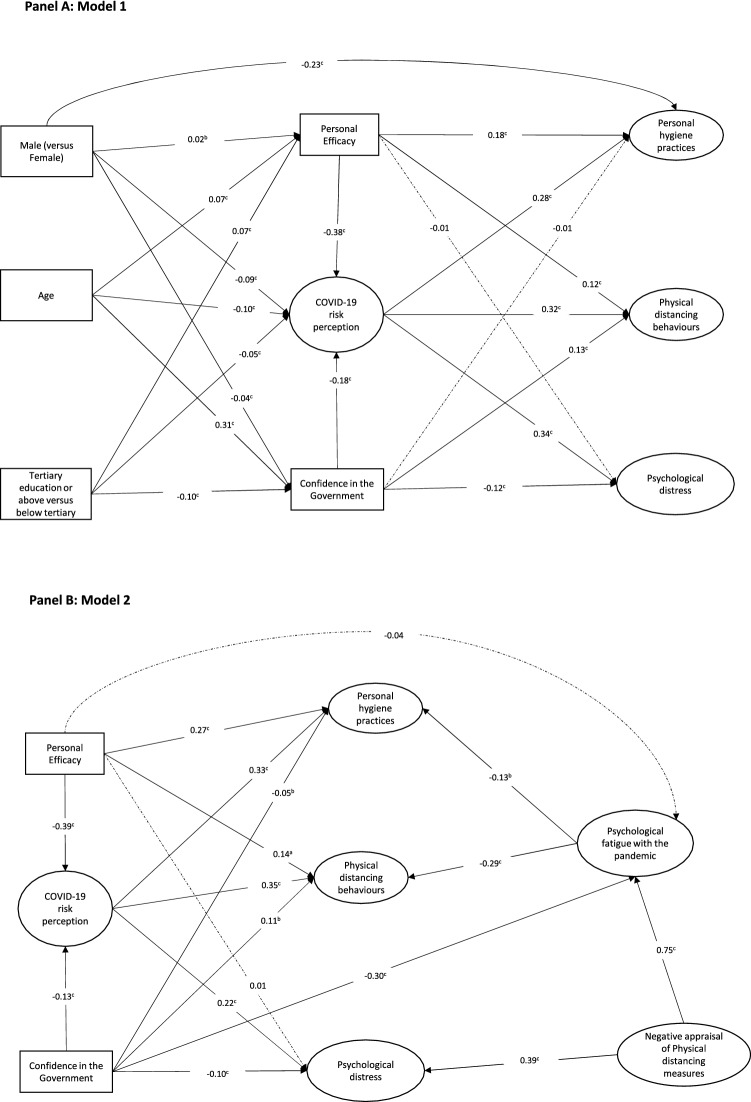
Associations among stress appraisal, precautionary behaviours and psychological distress and their associated factors based on structural equation modelling (SEM). Variables in Oval shape were treated as latent variables while variables in rectangles were treated as observed variables in SEM. All numbers on the line arrows were standardized path coefficients. Dotted lines indicate that the path coefficients were not statistically significant. ^a^*p* < 0.05; ^b^*p* < 0.01; ^c^*p* < 0.001.

In Model 1 (Panel A, Fig. 4), we found that higher personal efficacy (β =  − 0.38, SE = 0.009, *p* < 0.001) and confidence in government (β =  − 0.18, SE = 0.004 *p* < 0.001) was associated with lower risk perception of COVID-19 while higher risk perception of COVID-19 was associated with more adoption of PHP (β = 0.28, SE = 0.006, *p* < 0.001), more PDB (β = 0.32, SE = 0.004, *p* < 0.001) and greater psychological distress (β = 0.34, SE = 0.012, *p* < 0.001). Personal efficacy was positively associated with adoption of PHP (β = 0.18, SE = 0.003, *p* < 0.001) and PDB (β = 0.12, SE = 0.002, *p* < 0.001) while more confidence in government was associated with more PDB (β = 0.13, SE = 0.001, *p* < 0.001) and less psychological distress (β =  − 0.12, SE = 0.004, *p* < 0.001). In addition, we found that older individuals perceived lower risk from COVID-19 (β =  − 0.10, SE = 0.003, *p* < 0.001) and had more confidence in government’s pandemic control (β = 0.31, SE = 0.004, *p* < 0.001) while better educated individuals had lower confidence in government (β =  − 0.10, SE = 0.017, *p* < 0.001). Unemployment was initially included in Model 1 as a predictor of COVID-19 risk perception, personal efficacy and confidence in government but was not significantly associated with any of these variables and thereby was removed from the final model. In Model 2 (Panel B, Fig. 4), we found that more negative appraisal of physical distancing measures was associated with greater psychological distress (β = 0.39, SE = 0.029, *p* < 0.001), and greater psychological fatigue (β = 0.75, SE = 0.040, *p* < 0.001). Lower confidence in government was also associated with greater psychological fatigue (β =  − 0.30, SE = 0.010, *p* < 0.001) which in turn was associated with lower adoption of PHP (β =  − 0.13, SE = 0.026, *p* = 0.001) and PDB (β =  − 0.29, SE = 0.037, *p* < 0.001). Details of the SEM results are provided in Supplemntary Tables 2 and 3.

## Discussion

Our study showed that public worry about contracting COVID-19 changed in line with the community incidence of COVID-19 throughout the first year of the pandemic. Perceived susceptibility to COVID-19 was initially higher but declined thereafter and maintain at a low level. The discrepancy between worry about and perceived susceptibility to COVID-19 suggests that emotional response to a threat is more context- and value-driven rather than a mere reflection of cognitive estimate of probability of infection^32^. Emotional response to a hazard can be more heuristic and quicker to help people navigate during uncertain and complex situations while cognitive judgement of risk probability tends to be subject to biases^33,34^. The initially high perceived susceptibility to COVID-19 could be due to the high uncertainty of the disease^35,36^. As knowledge about the disease accumulates, cognitive judgement about risk could decline and remain at a relatively stable level^25^.

Both personal efficacy in self-protection and confidence in government’s ability to control the pandemic significantly declined when widespread of COVID-19 in the community was seen. Personal self-efficacy and confidence in the government were important determinants of adoption of precautionary behaviours^4,6^. Without sufficient confidence in self-protection or confidence in the government capacity to control the pandemic, public precautionary behaviours tend to be worry-driven, which could lead to helplessness to act^37^, unhealthy coping behaviours^38^, or avoidance of COVID-19 news^39^. In the context of a pre-existing low level of public trust in government in Hong Kong relating to major social unrest since June 2019^40^ and the historical experience of SARS in 2003–2004^41^, public trust in government appeared to quickly erode and difficult to recover, posing enormous challenges for the Hong Kong government for managing the pandemic.

While ‘behavioural fatigue’, one’s declining ability to perform behaviours over time due to complex underlying psychological mechanism, is questioned to be real^42^, the phenomenon of ‘pandemic fatigue’ is documented by the WHO^23^. Adding to the literature, our current study found that the population were able to maintain high compliance with most precautionary behaviours such as good hand hygiene, avoiding crowded places and avoiding social gathering throughout the first year of the pandemic but behaviours such as avoiding going outside, avoiding healthcare facilities, and avoiding non-essential use of public transport (e.g., for holidays) tend to relax when community incidence of COVID-19 declined. This indicates that the relaxation of some precautionary behaviours is not the result of the impaired ability to perform the tasks over time but the declining worry about COVID-19 and possibly the high cost of sustaining these behaviours^22^.

Another relative novel finding of our study was that negative appraisal of personal effort and its failure to achieve certain goals (pandemic control) as well as negative appraisal of the coping strategies (i.e., physical distancing measures) were associate with feeling of fatigue which in turn was associated with lower compliance with the precautionary behaviours. This highlights the importance of a self-regulatory feedback process to sustain compliance with challenging behaviours. Lacking confidence in government’s control measures may further deteriorate the feeling of fatigue. The study conducted among Italian in November 2020 also found that lower trust in government was associated symptoms of chronic fatigue which in turn was associated with reduced adoption of precautionary behaviours^43^, suggesting that the finding is robust across cultures. During a crisis, a trustworthy government can provide regular feedbacks (e.g., decline in transmission due to high public compliance with precautionary measures) for sustaining public’s precautionary behaviours^44^. Lacking such feedbacks for the great efforts put on compliance with the challenging behaviours could increase the feeling of ‘fatigue’.

The prevalence of probable anxiety and probable depression throughout the first year of the pandemic in Hong Kong was lower than the synthesized prevalence of anxiety and depression reported by recent systematic reviews^7–11^. This indicates that the pandemic may not have resulted in relatively higher substantial distress in the population in Hong Kong. Most people can maintain mentally healthy throughout the pandemic but a small group may suffer from “delayed dysfunction”^16^. Although the pandemic was the primary stressor that cause psychological distress, the interaction between the changing situation caused by the pandemic and individuals’ resources to cope with it was more important than the situation itself for individuals’ stress response^20^. Individuals who perceive more personal and government’s capacity to cope with the threat could shape less negative meaning of the situation which in turn mitigate their psychological distress. Confidence in government and believing that they have done what is needed for the best interest of public health are important to determine to what extent the public would tolerate and accept the challenging control measures^4,6,43^ and hence also has a direct psychological impact.

Our study found that female, younger, unemployed individuals, and individuals with lower educational attainment were more vulnerable to psychological distress. This is consistent with previous systematic reviews^8,10,11^. Adding to existing literature, our structural equation model indicates that females and less-educated individuals may report more anxiety symptoms throughout COVID-19 pandemic due to their greater risk perception of COVID-19 and lower personal efficacy in self-protection. Previous studies also reported that females compared with males were more concerned about the pandemic risk, had more negative emotional experience during the pandemic^45^, and had lower resilience in coping with the pandemic^46^. The gender disparity in health risk perception and capacity to cope with adversity could be attributed to biological, social and cultural factors^47^. However, gender disparity in depression was not indicated in our study, possibly because women’s job security and financial consequences may be affected less by the pandemic in Hong Kong^48^, which may somewhat mitigate their depressive symptoms.

Although older age was an important predictor of mortality due to COVID-19^49^, our SEM found that older adults perceived lower risk of COVID-19, perceived greater efficacy in self-protection and had more confidence in government’s pandemic control. The study conducted in the US also found that although older adults perceived greater risk of dying from COVID-19 if infected, they perceived lower risk of being infected and thereby less anxiety and depression (Wändi^50^. In contrast, young individuals aged 18–24 were found to have the least confidence in government in our study. In addition, younger adults may be more worried about being socially isolated if they were quarantined, feel more financial insecurity and perceive greater occupational difficulty during the pandemic (Wändi^50,51^. All these can contribute to the greater psychological distress in younger adults.

Unemployment has been well documented as a risk factor for psychological distress during the pandemic^11^. Adding to existing literature, our study found that the associations of unemployment with more anxiety and depression were persistently over one year of the pandemic. Unemployment was found to be not associated with risk perception of COVID-19, personal efficacy and confidence in government in our study. Possible reasons for why unemployment was persistently associated with greater psychological distress may be that unemployment impairs not only the financial capacity to cope with the stress but also sense of self-esteem, personal identity and social connections^52–54^. Unemployed individuals suffer from double stress from both the pandemic and losing jobs (or seeking jobs)^55^ and may have less access to insured medical services^56^. Future studies should be conducted to provide more comprehensive and in-depth assessment of how unemployment affects mental health during the pandemic.

Our study had some limitations. First, the cross-sectional nature of our data means that we are not able to examine the within-person changes of the psycho-behavioural responses throughout the pandemic and that the associations examined in our study may be attenuated by reverse causality. Second, sampling errors may generate differences in major study outcomes across survey rounds though each round had a relatively large sample size and all study outcomes were weighted by population age and sex. Third, some of our study constructs were assessed using single items, which means that these constructs may not be adequately assessed and thereby measurement errors may affect the reliability of parameter estimates. However, there are concerns over response rates and survey efficiency if long questionnaires were used. Fourth, the PHQ-4 used in our study was only an ultra-brief scale for screening possible anxiety and depression. However, its discriminative ability to detect a clinical diagnosis of anxiety or depression relative to a structured psychiatric interview was more than 0.75 measured with the area under the receiver operating characteristic curve^28,31^.

## Conclusions

Our study suggests that risk communication should focus on promoting the public’s confidence in self-protection and pandemic control as the pandemic resurges to avoid defensive response and feeling hopeless to act. Rebuilding public trust in the government should be a political priority to reduce psychological fatigue and psychological distress and sustain compliance with the precautionary behaviours. The government and relevant organizations should also provide practical support for the public to sustain adherence to more challenging behaviours and proper positive feedbacks and encouragement of their precautionary behaviours to reduce psychological fatigue during a long-lasting catastrophe. Furthermore, our study suggests that more social support should be provided for females, younger, less educated, and unemployed individuals to help them adapt to the stress caused by the pandemic. In particular, enhancing job security, preventing job loss and creating opportunity for re-employment should be put as one priority of policy making during the pandemic.

## Supplementary Information

Supplementary Information.

Supplementary Tables.
