# Supplementary material for: Community psychological and behavioural responses to coronavirus disease 2019 over one year of the pandemic in 2020 in Hong Kong
Source: Sci Rep. 2021 Nov 18;11:22480. doi: 10.1038/s41598-021-00616-9 (PMC8602294; doi:10.1038/s41598-021-00616-9)
Supplement: Supplementary file 2 — Supplementary Tables. [file 41598_2021_616_MOESM2_ESM.docx]

Supplementary Table 1 Descriptions of study measures

| Study measures | Items and response scales | Scale reliability (Cronbach’s α) | Score range | Mean (SD)^a^ |
| --- | --- | --- | --- | --- |
| **Core study measures** |  |  |  |  |
| Threat appraisal | RP1. Perceived likelihood of being infected | 0.52 | 1-7 | 3.33 (1.29) |
|  | RP2. Perceived severity of COVID-19 |  | 1-5 | 3.87 (0.93) |
|  | RP3. Worry about COVID-19 infection |  | 1-5 | 2.40 (1.06) |
| *Appraisal of control* | CON1. I’m confident that I can protect myself against COVID-19 (5-point agreement) | - | 1-5 | 3.74 (0.92) |
|  | CON2: I believe that the government of Hong Kong can take effective measure to control COVID-19 epidemic in Hong Kong | - | 1-5 | 2.73 (1.33) |
| *Physical distancing behaviours* | Over the past one week, did you (yes/no): |  |  |  |
|  | AV1. Avoid going to crowded places? | 0.71 | 0-1 | 0.85 (0.35) |
|  | AV2. Stay at home as much as possible? |  | 0-1 | 0.77 (0.42) |
|  | AV3. Avoid going to health care facilities? |  | 0-1 | 0.77 (0.42) |
|  | AV4. Avoid using public transport if unnecessary? |  | 0-1 | 0.88 (0.33) |
|  | AV5. Avoid social gathering? |  | 0-1 | 0.64 (0.48) |
|  | AV6. Maintain appropriate social distance with others outside of family? |  | 0-1 | 0.84 (0.37) |
| *Personal hygiene practices* | Over the past one week, how frequently did you (Never/sometimes/usually/always): |  |  |  |
|  | HY1. Use face mask when going outside? | 0.70 ^b^ | 1-4 | 3.94 (0.28) |
|  | HY2. Wash hands after going outside? |  | 1-4 | 3.76 (0.57) |
|  | HY3. Avoid direct touching public objects? |  | 1-4 | 2.66 (1.12) |
|  | HY4. Wash or disinfect hands after touching public objects immediately? |  | 1-4 | 3.11 (0.94) |
|  | HY5. Use liquid soap when washing hands? |  | 1-4 | 3.54 (0.73) |
|  | HY6. Rub hands for at least 20 seconds with soap when washing hands? |  | 1-4 | 3.12 (0.97) |
| *Psychological distress* | *Psychological distress*: GAD-2 and PHQ-2 were combined as the PHQ-4 scale to assess psychological distress | 0.78 | 0-12 | 1.71 (2.60) |
| **Additional study measures** |  |  |  |  |
| *Appraisal of the negative impacts of physical distancing measures* | NAP1. The social distancing measures have negatively affected my relationship with my friends. | 0.76 | 1-5 | 2.66 (1.17) |
|  | NAP2. The social distancing measures have negatively affected my family relationship. |  | 1-5 | 2.33 (1.11) |
|  | NAP3. Always staying at home makes me feel bored or isolated. |  | 1-5 | 2.91 (1.23) |
|  | NAP4. Staying alert to the risk of COVID-19 is stressful. |  | 1-5 | 2.84 (1.16) |
|  | NAP5.The social distancing measures have seriously disrupted my daily routines. |  | 1-5 | 2.95 (1.15) |
|  | NAP6. The social distancing measures have greatly interfered with my family income. |  | 1-5 | 2.85 (1.21) |
| *Psychological fatigue with COVID-19* | PF1. I’m tired of experts warning me about the risk of COVID-19. | 0.69 | 1-5 | 2.55 (1.11) |
|  | PF2. I feel tired or bored with information about COVID-19. |  | 1-5 | 2.63 (1.12) |
|  | PF3. I feel hopeless about ending the COVID-19 pandemic in Hong Kong because the virus will always be around. |  | 1-5 | 2.86 (1.20) |
|  | PF4. I feel frustrated about following COVID-19 protection guidelines because no matter how much I do, the pandemic may resurge |  | 1-5 | 2.95 (1.25) |

^a^ The mean and standard deviation (SD) of each item was calculated based on all available data across round.

^b^ This was calculated by excluding HY1 because wearing facemasks outside was compulsory in Hong Kong.

Supplementary Table 2 Details of the structural equation modelling results

| Model and parameter estimate |  | B | SE | β | p |
| --- | --- | --- | --- | --- | --- |
| **Model 1** |  |  |  |  |  |
| Factor loading |  |  |  |  |  |
| PSD to | PHQ1 | 1.000 | - | 0.806 | - |
|  | PHQ2 | 0.859 | 0.011 | 0.755 | <0.001 |
|  | PHQ3 | 0.881 | 0.011 | 0.791 | <0.001 |
|  | PHQ4 | 0.811 | 0.011 | 0.680 | <0.001 |
| PDB to | AV1 | 1.000 | - | 0.624 | - |
|  | AV2 | 1.222 | 0.019 | 0.639 | <0.001 |
|  | AV3 | 0.718 | 0.017 | 0.373 | <0.001 |
|  | AV4 | 0.745 | 0.015 | 0.497 | <0.001 |
|  | AV5 | 1.183 | 0.021 | 0.544 | <0.001 |
|  | AV6 | 0.972 | 0.017 | 0.581 | <0.001 |
| PHP to | HY2 | 1.000 | - | 0.523 | - |
|  | HY3 | 1.940 | 0.048 | 0.516 | <0.001 |
|  | HY4 | 2.052 | 0.046 | 0.651 | <0.001 |
|  | HY5 | 1.484 | 0.030 | 0.606 | <0.001 |
|  | HY6 | 2.008 | 0.044 | 0.619 | <0.001 |
| RP to | RP1 | 1.000 | - | 0.512 | - |
|  | RP2 | 0.526 | 0.015 | 0.372 | <0.001 |
|  | RP3 | 1.289 | 0.028 | 0.802 | <0.001 |
| *Path coefficient* |  |  |  |  |  |
| RP on | CON1 | -0.276 | 0.009 | -0.385 | <0.001 |
|  | CON2 | -0.089 | 0.004 | -0.179 | <0.001 |
|  | SEX | -0.115 | 0.009 | -0.087 | <0.001 |
|  | AGE | -0.040 | 0.003 | -0.101 | <0.001 |
|  | EDU | -0.061 | 0.010 | -0.046 | <0.001 |
| CON1 on | SEX | 0.029 | 0.011 | 0.016 | 0.007 |
|  | AGE | 0.040 | 0.004 | 0.074 | <0.001 |
|  | EDU | 0.130 | 0.012 | 0.070 | <0.001 |
| CON2 on | SEX | -0.110 | 0.015 | -0.041 | <0.001 |
|  | AGE | 0.244 | 0.004 | 0.312 | <0.001 |
|  | EDU | -0.274 | 0.017 | -0.103 | <0.001 |
| PHP on | CON1 | 0.057 | 0.003 | 0.177 | <0.001 |
|  | CON2 | -0.001 | 0.002 | -0.006 | 0.404 |
|  | RP | 0.125 | 0.006 | 0.278 | <0.001 |
|  | SEX | -0.139 | 0.005 | -0.233 | <0.001 |
| PDB on | CON1 | 0.029 | 0.002 | 0.120 | <0.001 |
|  | CON2 | 0.022 | 0.001 | 0.130 | <0.001 |
|  | RP | 0.105 | 0.004 | 0.317 | <0.001 |
| PSD on | CON1 | -0.007 | 0.007 | -0.009 | 0.321 |
|  | CON2 | -0.062 | 0.004 | -0.121 | <0.001 |
|  | RP | 0.345 | 0.012 | 0.337 | <0.001 |
| **Model 2** |  |  |  |  |  |
| Factor loading |  |  |  |  |  |
| PSD to | PHQ1 | 1.000 | - | 0.831 | - |
|  | PHQ2 | 0.880 | 0.033 | 0.789 | <0.001 |
|  | PHQ3 | 0.849 | 0.033 | 0.808 | <0.001 |
|  | PHQ4 | 0.800 | 0.041 | 0.683 | <0.001 |
| PDB to | AV1 | 1.000 | - | 0.603 | - |
|  | AV2 | 1.333 | 0.082 | 0.652 | <0.001 |
|  | AV3 | 0.657 | 0.064 | 0.346 | <0.001 |
|  | AV4 | 0.649 | 0.052 | 0.458 | <0.001 |
|  | AV5 | 1.218 | 0.075 | 0.558 | <0.001 |
|  | AV6 | 0.992 | 0.064 | 0.562 | <0.001 |
| PHP to | HY2 | 1.000 | - | 0.544 | - |
|  | HY3 | 1.651 | 0.154 | 0.494 | <0.001 |
|  | HY4 | 1.831 | 0.150 | 0.639 | <0.001 |
|  | HY5 | 1.491 | 0.100 | 0.642 | <0.001 |
|  | HY6 | 1.923 | 0.148 | 0.658 | <0.001 |
| RP to | RP1 | 1.000 | - | 0.530 | - |
|  | RP2 | 0.518 | 0.059 | 0.370 | <0.001 |
|  | RP3 | 1.064 | 0.095 | 0.745 | <0.001 |
| NAP to | NAP1 | 1.000 | 0 | 0.632 | - |
|  | NAP2 | 0.867 | 0.038 | 0.581 | <0.001 |
|  | NAP3 | 1.024 | 0.049 | 0.616 | <0.001 |
|  | NAP4 | 0.981 | 0.050 | 0.629 | <0.001 |
|  | NAP5 | 0.946 | 0.044 | 0.607 | <0.001 |
|  | NAP6 | 0.758 | 0.047 | 0.464 | <0.001 |
| PF to | PF1 | 1.000 | 0 | 0.420 | - |
|  | PF2 | 1.236 | 0.074 | 0.513 | <0.001 |
|  | PF3 | 1.514 | 0.122 | 0.587 | <0.001 |
|  | PF4 | 1.896 | 0.141 | 0.706 | <0.001 |
| *path coefficient* |  |  |  |  |  |
| RP on | CON1 | -0.300 | 0.044 | -0.392 | <0.001 |
|  | CON2 | -0.070 | 0.015 | -0.134 | <0.001 |
| PHP on | CON1 | 0.098 | 0.014 | 0.270 | <0.001 |
|  | CON2 | -0.013 | 0.007 | -0.051 | 0.003 |
|  | RP | 0.156 | 0.025 | 0.330 | <0.001 |
|  | PF | -0.090 | 0.026 | -0.127 | 0.001 |
| PDB on | CON1 | 0.034 | 0.009 | 0.137 | <0.001 |
|  | CON2 | 0.019 | 0.006 | 0.110 | 0.003 |
|  | RP | 0.114 | 0.017 | 0.349 | <0.001 |
|  | PF | -0.143 | 0.037 | -0.292 | <0.001 |
| PSD on | CON1 | 0.008 | 0.020 | 0.012 | 0.667 |
|  | CON2 | -0.050 | 0.011 | -0.105 | <0.001 |
|  | RP | 0.199 | 0.037 | 0.219 | <0.001 |
|  | NAP | 0.333 | 0.029 | 0.393 | <0.001 |
| PF on | CON1 | -0.023 | 0.013 | -0.045 | 0.068 |
|  | CON2 | -0.106 | 0.010 | -0.302 | <0.001 |
|  | NAP | 0.465 | 0.040 | 0.747 | <0.001 |

PSD: psychological distress; PDB: physical distancing behaviours; PHP: personal hygiene practices; RP: COVID-19 risk perception; CON1: personal efficacy; CON2: confidence in government’s pandemic control; NAP: negative appraisal of physical distancing measures; PF: psychological fatigue with the pandemic; PH1-4: indicators of psychological distress; AV1-AV6: indicators of physical distancing behaviours; HY2-6: indicators of personal hygiene practices; NAP1-6: indicators of negative appraisal of physical distancing measures; PF1-4: indicators of psychological fatigue with the pandemic. SEX: sex of the participants coded as 1=male and 0=female; AGE: age groups of the participants coded as 0=18-24 years, 1=25-34 years, 2=35-44 years, 3=45-54 years, 4=55-64 years and 5=65 years or above; EDU: educational attainment of the participants coded as 1= tertiary education or above and 0= secondary education or below.

In Model 1, unemployment was initially included as a predictor of RP, CON1 and CON2 but was not significantly associated with any variables of stress appraisal and thereby was removed from the final model. A path from SEX to PHP was additionally suggested by the model modification indices.

Supplementary Table 3 Model fit indices of the models tested using structural equation modelling

| Model | CFI | TFI | SMRS | RMSEA (90% CI) | Sample size |
| --- | --- | --- | --- | --- | --- |
| Model 1 | 0.926 | 0.911 | 0.033 | 0.036 (0.035-0.036) | 3,095 |
| Model 2 | 0.926 | 0.917 | 0.044 | 0.034 (0.032-0.037) | 1,972 |
